# Supplementary material for: Fast Detection of 10 Cannabinoids by RP-HPLC-UV Method in Cannabis sativa L
Source: Molecules. 2019 Jun 4;24(11):2113. doi: 10.3390/molecules24112113 (PMC6600594; doi:10.3390/molecules24112113)
Supplement: Supplementary file 1 [file molecules-24-02113-s001.zip › molecules-513726-supple-proofed/Supplementary material File 1 SOP.pdf]

File 1 SI. Standard operating procedure (SOP) of the method presented in this article

**EDITOR**	Chemical expert Mara Mandrioli
**PROCEDURE MANAGER**	Professor Tullia Gallina Toschi
**VERIFICATION**	SPP
**APPROVAL**	
**AUTHORIZATION**	

STATUS OF REVISIONS

**REV. N.**	**SUBJECT OF THE REVISION**	**DATE**
**0**	First draft	2018/11/26
**1**	Revisioned by …………………	……………….

LIST OF ANNEXES

**CODE/ACRONYM**	**NOME**
Annex 1	Chromatogram of a solution of analytical reference standards.

1. PURPOSE

he Italian Ministry of Health, with the enactment of Legislative Decree No. 36/2014 published in the Official Gazette of 20/05/14, No. 67, introduced cannabis-based medicines of plant origin: leaves, inflorescences, oleolites and dyes in table II sec. B of narcotic and psychotropic substances allowing the possibility of preparing masterly preparations. The therapeutic effect of cannabis-based extract-preparations is a direct consequence of their composition and of the titre of the different cannabinoid, which are the active ingredients. In order to obtain titrated and safe quality extract-preparations, their rigorous quali-quantitative analytical evaluation is essential. Moreover, the evaluation and the monitoring of the content of cannabinoid, during the cultivation and storage phases of hemp, guarantees product uniformity and optimization of production yields. The present procedure, drawn up following the guidelines indicated by ISO 78-2, ISO 5725 and the regulation Reg. 2017/625, aims to determine the quali-quantitative profile of the cannabinoids present in the Cannabis inflorescences.

This document is property of DISTAL and may not be used, reproduced or made known to third parties without authorization from the MANAGEMENT

2. FIELD OF APPLICATION

The Operating Procedure is applied, with reference to the laws in force, for the adoption and compliance with the organizational criteria introduced for:

- Worker health and safety protection;
- The management organization of the aspects of security within the Plexus;
- The application of the concepts of Good Practice in laboratory activities;
- The application of Quality / Safety standards in research activities.

3. PLACE OF APPLICATION

The procedure described is applied within the DISTAL Food Evaluation and Analysis Laboratory located at the DISTAL in Viale Fanin, 40 on the fifth floor, west wing.

ABBREVIATIONS, DEFINITIONS, TERMINOLOGY, SYMBOLOGY

**ABBREVIATIONS**
**CBD**, Cannabidiol; **CBG**, Cannabigerol; **Δ9-THC**, Δ9- Tetrahydrocannabinol; **CBN**, Cannabinol; **THCV**, Tetrahydrocannabivarin; **CBC**, Cannabicromene; **CBDA**, Cannabidiolic Acid; **CBGA**, Cannabigerolic Acid; **Δ8-THC**, Δ8- Tetrahydrocannabinol; **THCA**, Tetrahydrocannabinolic acid; **HPLC**, High Performance Liquid Chromatography; **UV**, ultraviolet.
**DEFINITIONS AND TERMINOLOGY**

**SYMBOLOGY**	
P	It highlights a **dangerous** condition for the health and safety of the operator
⚠	It highlights **Attention / Recommendation** in the way of working **(critical point)**

4. REGULATORY AND DOCUMENT REFERENCES

DECREE-LAW of March 20, 2014, n. 36. "Disposizioni urgenti in materia di disciplina degli stupefacenti e sostanze psicotrope, prevenzione, cura e riabilitazione dei relativi stati di tossicodipendenza, di cui al decreto del Presidente della Repubblica 9 ottobre 1990, n. 309, nonché

This document is property of DISTAL and may not be used, reproduced or made known to third parties without authorization from the MANAGEMENT

di impiego di medicinali meno onerosi da parte del Servizio sanitario nazionale." OFFICIAL JOURNAL OF THE ITALIAN REPUBLIC (GAZZETTA UFFICIALE DELLA REPUBBLICA ITALIANA Serie generale - n. 67).

Reg.1155/2017 of February 15th, 2017 which amends the delegated regulation (UE) n. 639/2014 as regards the control measures relating to the cultivation of hemp, some provisions relating to the payment of greening, to the payment for young farmers exercising control over a legal person, to the calculation of the unit amount under the optional coupled support, to fractions of payment entitlements and certain reporting obligations relating to the single area payment scheme and voluntary coupled support, and which amends Annex X of the Regulation (UE) n. 1307/2013.

De Backer D.B., Debrus B. and Lebrun P.; Innovative development and validation of an HPLC/DAD mathod for the qualitative and quantitative determination of major cannabinoids in cannabis plant material; Journal of Chromatography B: Analytical Technologies in the Biomedical and Life Sciences, 877(32): 4115-4124, 2009.

5. FASI DEL PROCESSO E DIAGRAMMA DI FLUSSO DELLE ATTIVITA'

FASE	INPUT	ACTIVITIES	OUTPUT
1		BEGINNING	
2		DRYING AND STORAGE OF SAMPLES	
3		EXTRACTION	
4		SAMPLE COLLECTION	
5		PREPARATION OF STANDARD SOLUTIONS	
6		CHROMATOGRAPHIC ANALYSIS	
...		END	

6. LIST OF THE PRODUCTS / REAGENTS USED

PHASE	PRODUCT	PREPARATION	SECURITY INSTRUCTIONS
1	Start of activity		

This document is property of DISTAL and may not be used, reproduced or made known to third parties without authorization from the MANAGEMENT

**2**	**Solution methanol:chloroform (9:1, v/v)**	Mix 9 parts of methanol and 1 part of chloroform (v/v).	**Chloroform CAS N.67-66-3**<br>Risk phrases: R 22-38-40-48/20/22. Harmful if swallowed.<br>rritating to the skin. Possible irreversible effects. Harmful: danger of serious damage to health by prolonged exposure through inhalation and ingestion.<br>Safety advice: S 36/37. Wear suitable protective clothing and gloves.<br>**Methanol CAS N. 67-56-1**<br>Risk phrases: R 11-23/24/25-39/23/24/25. Easily flammable. Toxic by inhalation, by ingestion and skin contact. Toxic: danger of very serious irreversible effects through inhalation, in contact with skin and if swallowed.<br>Safety advices: S 7-16-36/37-45. Keep the container tightly closed and away from flames and sparks. Do not smoke. Wear suitable protective clothing and gloves. In case of accident or discomfort, consult a doctor immediately.
**3**	**Nitrogen**<br>**CAS N. 07727-37-9**	Gas prepared.	High concentrations asphyxiant. **Safety advices:** S 9-23. Keep the container in a well-ventilated place. Do not breathe the gas. Work under the extractor hood.
**4**	**Acetonitrile for chromatography**<br>**CAS N : 75-05-8**	Solvent prepared	Indications of danger:<br>H225 Highly flammable liquid and vapor.<br>H302+H312+H332 Harmful if swallowed. Harmful in contact with skin. Harmful if inhaled.<br>H319 It causes serious eye

This document is property of DISTAL and may not be used, reproduced or made known to third parties without authorization from the MANAGEMENT

			irritation.<br>Safety advices:<br>P210 Keep away from heat / sparks / open flames / heated surfaces. Do not smoke.<br>P280 Wear protective gloves / protective clothing / eye protection / face protection.<br>P305+P351+P338 IN CASE OF EYES CONTACT: Rinse thoroughly for several minutes. Remove any contact lenses if it is easy to do so. Continue to rinse.<br>P309+P310 IN CASE of exposition or of illness: Contact a POISON CENTER or a doctor immediately.
**5**	**Phosphoric acid 85% v/v**<br>**N.CAS : 7664-38-2**	0.085% solution in pure water for chromatography.<br><br>0.085% solution in pure acetonitrile for chromatography.	Indications of danger:<br>H314 It causes serious skin burns and serious eye injuries.<br>Safety advices:<br>P280 Wear protective gloves / protective clothing / eye protection / face protection.
**6**	**Pure water for chromatography**<br>**CAS N : 7732-18-5**	Solvent prepared.	Solvent not dangerous according to CE Regulation N.1272 / 2008 and Directive 67/548 / CEE.
**7**	**Cannabinoid standard solution**	Prepare solutions of known concentration by diluting the standard mixture using acetonitrile as a solvent.	Acetonitrile solution 99.9 %<br>CAS # /RTECS # 75-05-8<br>H225<br>H302<br>H312<br>H319<br>H332<br>**Cannabidiolic Acid 0.01 %**<br>CAS # /RTECS # 1244-58-2<br><br>**Cannabigerolic Acid 0.01 %**<br>CAS # /RTECS # 25555-57-1

This document is property of DISTAL and may not be used, reproduced or made known to third parties without authorization from the MANAGEMENT

			**Cannabigerol 0.01 %**<br>CAS # /RTECS # 25654-31-3<br><br>**Cannabidiol 0.01 %**<br>CAS # /RTECS # 13956-29-1<br>H301<br>H361<br><br>**Tetrahydrocannabivarin 0.01 %**<br>CAS # /RTECS # 31262-37-0<br>H400<br>H410<br><br>**Cannabinol 0.01 %**<br>CAS # /RTECS # 521-35-7<br>H361<br><br>**THCA-A 0.01 %**<br>CAS # /RTECS # 23978-85-0<br>H302<br>H361<br><br>**Δ9-THC 0.01 %**<br>CAS # /RTECS # 1972-08-3<br>H302<br>H361<br><br>Δ8-THC 0.01 %<br><br>CAS # /RTECS # 5957-75-5<br>H302<br><br>Cannabichromene 0.01 %<br>CAS # /RTECS # 20675-51-8<br>Highly flammable liquid and vapor. Toxic if swallowed. Harmful in contact with skin. Causes serious eye irritation. Harmful if inhaled. Keep away from heat, hot surfaces, sparks, flames and other ignition sources. No smoking. Avoid breathing dust / fumes / gases / mist / vapors / aerosols. Wear protective gloves / clothing / eye protection / face

This document is property of DISTAL and may not be used, reproduced or made known to third parties without authorization from the MANAGEMENT

			protection. IF SWALLOWED: contact a POISON CENTER / doctor / ... / in case of malaise IN CASE OF CONTACT WITH SKIN: wash thoroughly with water. IN CASE OF CONTACT WITH SKIN (or hair): Remove contaminated clothing. Rinse the skin / take a shower. IN THE EVENT OF INHALATION, transport the injured person to fresh air and keep him in position which encourages breathing. IN CASE OF CONTACT WITH THE EYES. Rinse thoroughly for several minutes. Remove any contact lenses if it is easy to do so. Continue to rinse. If eye irritation persists, consult a doctor. Remove contaminated clothing and wash them before wearing them again.
**...**	**End of activity**		

7. DESCRIPTION OF THE PROCESS

PHASE	DESCRIPTION	IDENTIFICATION OF DANGERS	SAFETY INSTRUCTIONS
**1**	**Start of activity**		
**2**	**Sample drying and conservation.**<br>Place the sample in a stove at a temperature of 32°C for a period of 60 hours, until a constant weight is reached. Remove coarse parts like stems, seeds and leaves, trying to preserve the apical tops. Proceed to grinding using an analytical mill, avoid overheating of the plant material. The powder obtained is sieved with a sieve with a mesh width of 1 mm. Store the sample away from light and moisture at a temperature below 25°C for 10 weeks.	High temperature equipment. Risk of burns. Electrical equipment. Risk of electrocution injury. Risk of cutting wounds.	Check the temperature set on the stove.<br>Handle the mill blades with care and follow the instructions in the manual. Check the integrity of the power cables.
**3**	**Extraction**<br>Weigh an aliquot of pulverized sample 25 mg ± 0.1 in a glass tube.	Electrical equipment. Risk of electrocution injury. Use of centrifuge	Check the integrity of the power cables. Balance the volumes

This document is property of DISTAL and may not be used, reproduced or made known to third parties without authorization from the MANAGEMENT

	Add 10 mL of methanol-chloroform extraction solvent 9: 1 (v / v). Place for 10 min. on a tilting agitator set at 350 oscillations per minute, then 10 minutes in an ultrasonic bath. Centrifuge for 5 minutes at 1620 rpm and remove the supernatant. Repeat the extraction twice in the same way. Collect the two fractions containing the cannabinoid active ingredients in a 25 mL graduated flask and make up to volume with methanol / chloroform (9:1, v/v).	and tilting agitator with moving parts. Risks from high speed of moving parts. Use of glassware. Risk of cutting wounds. Use of hazardous solvents.	in the centrifuge and in the tilting agitator.<br>Follow the instructions of the manual. Wear safety glasses.<br>Chloroform CAS N.67-66-3<br>Risk phrases: R 22-38-40-48/20/22. Harmful if swallowed. Irritating to the skin. Possible irreversible effects. Harmful: danger of serious damage to health by prolonged exposure through inhalation and ingestion.<br>Safety advices: S 36/37. Wear suitable protective clothing and gloves.<br>Methanol CAS N. 67-56-1<br>Risk phrases: R 11-23/24/25-39/23/24/25. Easily flammable. Toxic by inhalation, by ingestion and skin contact. Toxic: danger of very serious irreversible effects through inhalation, in contact with skin and if

This document is property of DISTAL and may not be used, reproduced or made known to third parties without authorization from the MANAGEMENT

			swallowed.<br>Safety advices: S 7-16-36/37-45. Keep the container tightly closed and away from flames and sparks - Do not smoke. Wear suitable protective clothing and gloves. In case of accident or discomfort, consult a doctor immediately. Work under the extractor hood. Check the correct water level inside the ultrasonic bath.
**4**	**Sample collection**<br>Filter with a 45 µm nylon filter. Take 2 mL of filtered extract in a glass tube. Remove the solvent, bringing to dry with the aid of a weak nitrogen flow. Resume with 500 µL of acetonitrile. Transfer to vial for autosampler.	Use of glassware. Risk of cutting wounds. Use of hazardous solvents.	See point 3.
**5**	**Reference standard solutions.**<br>The quantification of the analytes is carried out using the external standard method, through the construction of calibration curves by using the standard reference mixture containing 10 analytes of chromatographic purity. Take aliquots of the reference solution to obtain diluted solutions in the required concentration range.	Use of glassware. Risk of cutting wounds. Use of hazardous solvents.	Perform all operations under the suction hood. Use nitrile protective gloves.
**6**	**HPLC-UV chromatographic analysis.**<br>Eluent mixture: Water + 0.1% phosphoric acid, B acetonitrile + 0.1%	Electrical equipment. Risk of electrocution injury. Use of glassware. Risk of cutting wounds.	Prepare the eluent mixture under the suction hood. Place the bottles in the

This document is property of DISTAL and may not be used, reproduced or made known to third parties without authorization from the MANAGEMENT

	phosphoric acid. Elution with gradient: 70% of B up to 3.0 min. 85% of B up to 7 min. 95% of B from 7.01 to 8.00 min. maintained up to 3.5 min. 70% B up to 10 min .;<br>Column temperature: 35 ° C.<br>Injection: 5.0 µl.<br>Shim-pack XR-ODS II column 75 x 3.0 mm, 2.2 µm.<br>Detector DAD: λ 220 nm.	Use of hazardous acid solvents.	special compartment using safety caps. Check the integrity of the power cables. Follow the instructions in the equipment manual
...			
...	**End of activity**		

8. OCCASIONAL AND / OR MAINTENANCE INTERVENTIONS OF THE INSTRUMENTATION

PHASE	DESCRIPTION	IDENTIFICATION OF DANGERS	SAFETY INSTRUCTIONS
1	**Start of activity**		
2	Check the correct operation of all the equipment following the calibration procedures where provided. Keep the equipment clean.	Electrical equipment that involves electrocution risks.	During routine and extraordinary cleaning and maintenance operations, where it is necessary, disconnect the power cables.
3	Accidental spills	Inhalation of toxic substances.<br>Risk of falling due to slipping.	In the case of accidental spillage of chloroform, methanol, near live equipment or at high temperatures, lower the glass of the hood, disconnect the power, leave at a safe distance taking care to warn all the operators present in the area, go towards the fire extinguisher. If spillage

This document is property of DISTAL and may not be used, reproduced or made known to third parties without authorization from the MANAGEMENT

			takes place away from equipment, collect it using FIRESORB flame-retardant universal absorbent material and dispose of the material safely. If spillage occurs on the floor, try to contain the spill with the same material, notify the workers and proceed with cleaning.
**4**	Waste disposal	Inhalation of toxic substances. Dispersion of polluting materials in the environment.	iltration residues, nylon and matrix filters, after drying under the hood, must be disposed of with waste from the Filter Absorbing Materials Code CER 150202. Residues from chromatographic elution (HPLC-UV) they must be disposed of with non-chlorinated organic waste Code CER 160508.<br>The residues deriving from the extraction must be disposed of with chlorinated organic waste Code CER 070103.<br>Any empty containers must be disposed of with empty container waste Code CER 150110.
**5**	Decontamination	No special decontamination procedures need to be developed.	Wash the glassware with water, soap and bottle brush. The wet glassware with organic solvents is left to dry under the hood before washing.
**...**	**End of activity**		

This document is property of DISTAL and may not be used, reproduced or made known to third parties without authorization from the MANAGEMENT

9. RESPONSIBILITY MATRIX

PHASE OF THE PROCESS / Function [(1)]	R.D.R.L.	Person in charge	Collaborator	Other
**1 Start of activity**				
1. Definition of the operational phases of the SOP	**R**	**C**		
2. Analytical description of each phase	**R**	**C**		
3. Waste management	**R**	**R**		
4. Document management	**R**	**C**		
**...**				
**... End of activity**				

[(1)] **Type of responsibility of the Functions involved in the process.**
R = Responsible **C** = Involved **NC** = Not involved **SR** = Deputy of the Responsible **CR** = Responsibility sharing

10. DOCUMENT MANAGEMENT

PHASE	INPUT	ACTIVITY
**DISTRIBUTION** 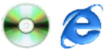	Ensure the knowledge and availability of the procedure for all collaborators and / or people in charge of the laboratory activity.	The procedure is made available and brought to the attention of all collaborators, through distribution, having read the paper and / or computer copy. for this purpose it is delivered to the knowledge of each collaborator at the time of taking up the service. The delivery and / or knowledge of the Procedure by the personnel is documented and regulated by a specific procedure established for the training and education of collaborators.
**STORAGE** 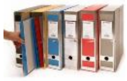	Collect and preserve the paper copy of the original procedure and forms, both in the Laboratory Security documents and in the "Department Security Manual" (Cap.8 - Technical Procedures).	The procedure is an integral part of the "Safety Manual of the Department" and is available to all collaborators who work within the Laboratory for its use and for their timely information at any time of the activity. The documents are kept in the original for a period of at least 5 years. The computer version is also stored on the security server.
**TRASMISSION** 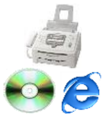	When it is requested, send the document to other interested users and / or offices, which, for different reasons, request it.	The procedure, being property of the Department, is forwarded to the interested parties on specific and motivated request. The document is sent to the applicants in paper and / or electronic form with prior authorization and with the obligation not to use

This document is property of DISTAL and may not be used, reproduced or made known to third parties without authorization from the MANAGEMENT

		it for personal purposes.

Annex 1 **Chromatogram of a solution of analytical reference standards.**

This document is property of DISTAL and may not be used, reproduced or made known to third parties without authorization from the MANAGEMENT
