# Supplementary material for: Fast Detection of 10 Cannabinoids by RP-HPLC-UV Method in Cannabis sativa L
Source: Molecules. 2019 Jun 4;24(11):2113. doi: 10.3390/molecules24112113 (PMC6600594; doi:10.3390/molecules24112113)
Supplement: Supplementary file 1 [file molecules-24-02113-s001.zip › molecules-513726-supple-proofed/Supplementary material File 2.pdf]

Preliminary tests carried out for the development of the analytical procedure by RP-HPLC-UV:

The following are some of the preliminary analysis that have been performed by testing different eluent mixtures, columns and chromatographic conditions.

TEST 1

HPLC system consisted of a Series 1260 chromatograph coupled to a Series 1100 autosampler and Diode Array Detector (DAD, Agilent Tecnologies, Palo Alto, CA) and Software HPLC ChemStation (Rev.A.08.03 Agilent Technologies, USA).

Eluent mixture: A water + 0.1% formic acid, B methanol-acetonitrile 75:25 + 0.1% formic acid.

Isocratic elution with Sphereclone 5µ ODS(2) 80 Å at a flow rate of 1.5 mL/min.

With these chromatographic conditions there is no acceptable separation of CBGA, CBG and there is the co-elution of CBD and THCV, as shown in figure below.

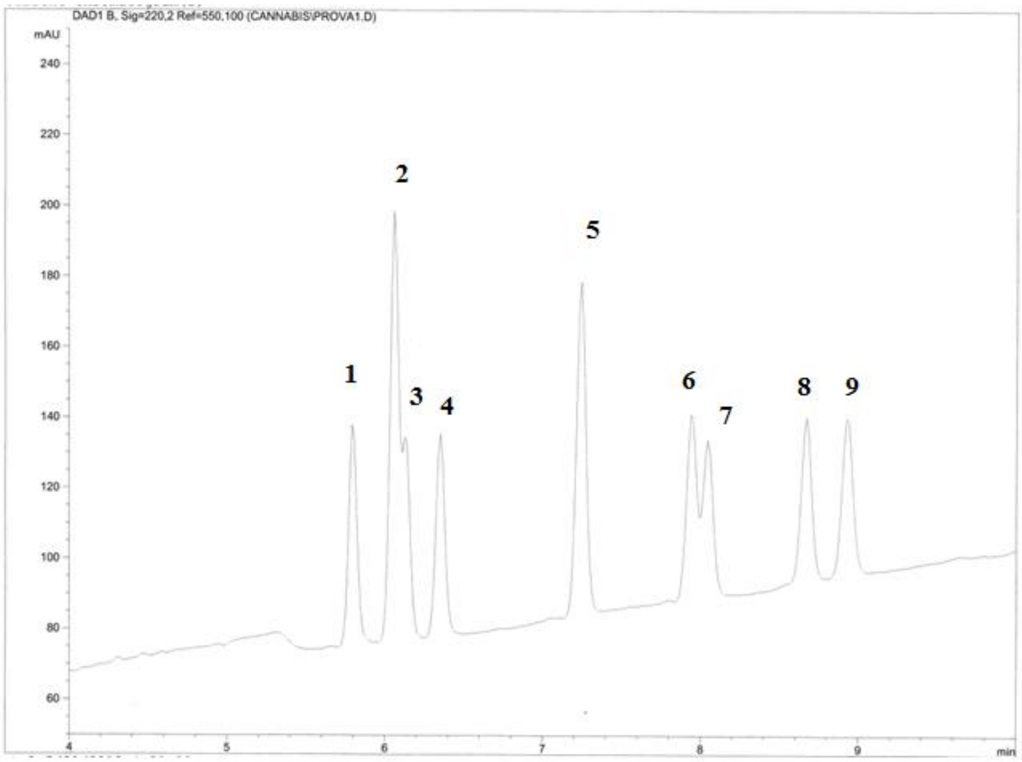

1-CBDA, 2-CBGA, 3-CBG, 4-CBD+THCV, 5-CBN, 6- Δ9- THC, 7- Δ8-THC, 8-CBC, 9THCA.

TEST 2

HPLC system consisted of a Series 1260 chromatograph coupled to a Series 1100 autosampler and Diode Array Detector (DAD, Agilent Tecnologies, Palo Alto, CA) and Software HPLC ChemStation (Rev.A.08.03 Agilent Technologies, USA).

Gradient elutions was applied with a flow rate of 1.0 mL/min., according to the following procedure.

Eluent mixture: A water + 0.1% phosphoric acid, B acetonitrile + 0.1% phosphoric acid.

Gradient elution with a Kinetex C18 150 x 4,6 mm., 2,6 µm 100 Å (Phenomenex) thermostatically at 50 °C, flow rate of 1.8 mL/min.

Gradient elution: 75% of B up to 6.0 min. 100% of B to 6.01, 75% of B maintained for 4 minutes.

With these chromatographic conditions all compounds are separated but there is no good separation of CBGA, CBG and Δ9- THC, Δ8-THC, as shown in the figure below.

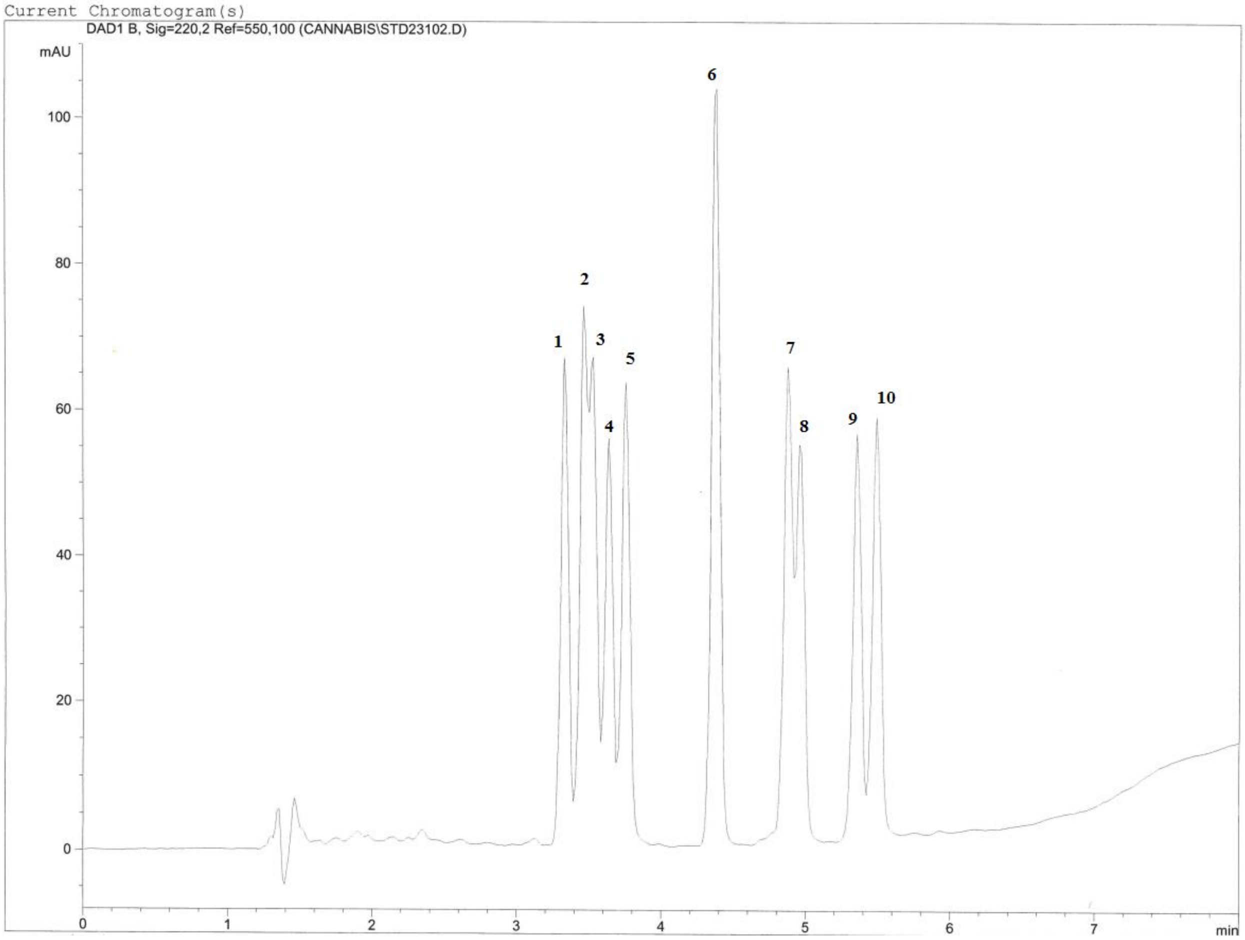

1-CBDA, 2-CBGA, 3-CBG, 4-CBD, 5THCV, 6-CBN, 7- Δ9- THC, 8- Δ8-THC, 9-CBC, 10-THCA

TEST 3

HPLC system consisted of a Cannabis Analyzer for Potency Prominence-i LC-2030C equipped with a reverse phase C18 column, Nex-Leaf CBX Potency 150 x 4.6 mm, 2.7 µm with a guard column Nex-Leaf CBX 5 x 4.6 mm, 2.7, UV detector and an acquisition software LabSolutions version 5.84 (Shimazu, Kyoto, Japan).

Gradient elution was used at flow rate of 1.5 mL/min., according to the following procedure.

Eluent mixture: A water + 0.1% phosphoric acid, B acetonitrile + 0.1% phosphoric acid.

Gradient elution: 75% of B up to 0.70 min. 85% of B to 2 min. 100% of B to 3.00 until 3.50 min. up to 3.60 min. and 75% of B up to 5 minutes.

With these chromatographic conditions there is no acceptable separation of CBDA, CBGA, CBG and there is the co-elution of CBD and THCV, as shown in figure below.

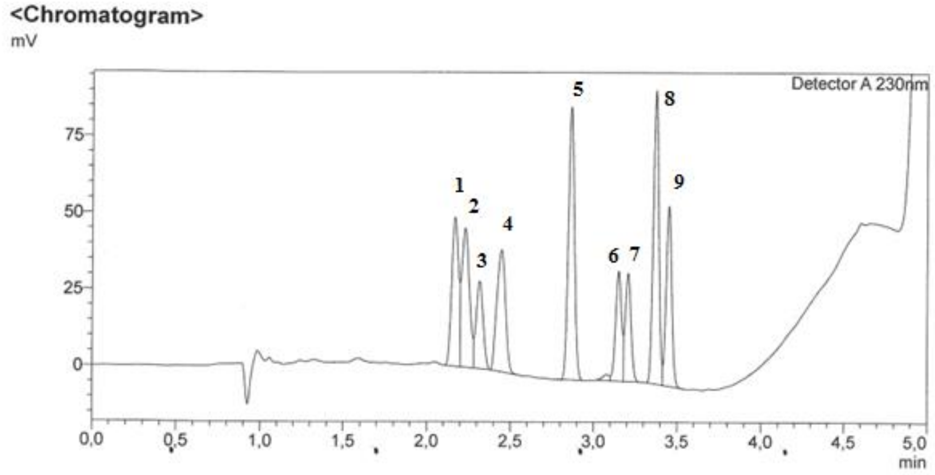

1-CBDA, 2-CBGA, 3-CBG, 4-CBD+THCV, 5-CBN, 6- Δ9- THC, 7- Δ8-THC, 8-CBC, 9-THCA.

Evaluation of the DAD detector response at different wavelengths 210, 220, 228, 273 nm.

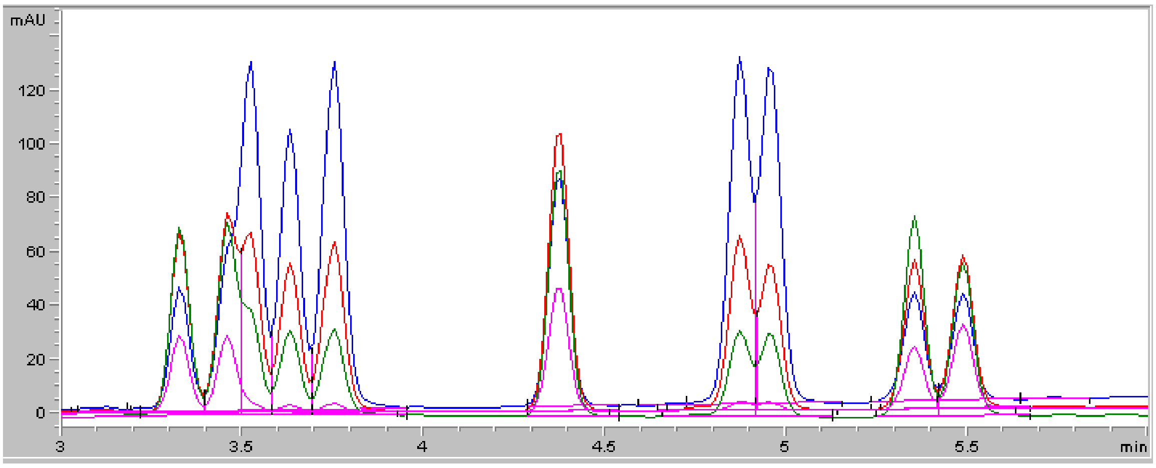

DAD1 A, Sig=210,2 Ref=550,100

DAD1 B, Sig=220,2 Ref=550,100

DAD1 C, Sig=228,2 Ref=550,100

DAD1 D, Sig=273,2 Ref=550,100
